# Structure and substrate selectivity of the 750-kDa α_6_β_6_ holoenzyme of geranyl-CoA carboxylase

**DOI:** 10.1038/ncomms9986

**Published:** 2015-11-23

**Authors:** Ashley R. Jurado, Christine S. Huang, Xing Zhang, Z. Hong Zhou, Liang Tong

**Affiliations:** 1 Department of Biological Sciences, Columbia University, New York, New York 10027, USA; 2 California NanoSystems Institute, University of California, Los Angeles, California 90095, USA; 3 Department of Microbiology, Immunology and Molecular Genetics, University of California, Los Angeles, California 90095, USA

## Abstract

Geranyl-CoA carboxylase (GCC) is essential for the growth of *Pseudomonas* organisms with geranic acid as the sole carbon source. GCC has the same domain organization and shares strong sequence conservation with the related biotin-dependent carboxylases 3-methylcrotonyl-CoA carboxylase (MCC) and propionyl-CoA carboxylase (PCC). Here we report the crystal structure of the 750-kDa α_6_β_6_ holoenzyme of GCC, which is similar to MCC but strikingly different from PCC. The structures provide evidence in support of two distinct lineages of biotin-dependent acyl-CoA carboxylases, one carboxylating the α carbon of a saturated organic acid and the other carboxylating the γ carbon of an α-β unsaturated acid. Structural differences in the active site region of GCC and MCC explain their distinct substrate preferences. Especially, a glycine residue in GCC is replaced by phenylalanine in MCC, which blocks access by the larger geranyl-CoA substrate. Mutation of this residue in the two enzymes can change their substrate preferences.

## Abstract (web summary)

The structures of the biotin-dependent carboxylases have revealed details of their function. Here, the authors describe the first structure of *Pseudomonas* geranyl-CoA carboxylase, and compare it with the previously characterised homologous 3-methylcrotonyl-CoA carboxylase.

## Graphical abstract

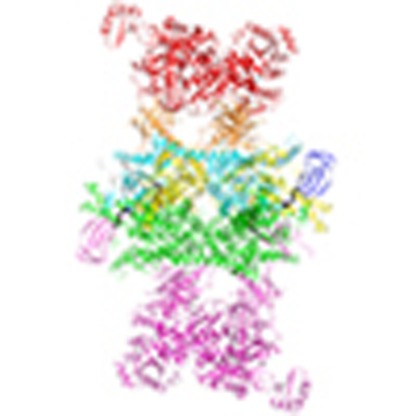

Terpenes are a common biosynthetic precursor of many biological compounds, including hormones and cholesterol, and have long been studied for their many medicinal properties^1^,^2^,^3^. Monoterpenes are the simplest terpenes and are formed by the joining of two five-carbon isoprene units. Terpenes are modified and cyclized in various ways to form numerous terpenoids, including hormone precursors, scents, and flavourings. Isoprenylation is also used for localizing proteins to the cell membrane. In certain bacteria, terpenes can be used as the primary carbon source and metabolized to enter the tricarboxylic acid cycle as acetyl-CoA.

Geranyl-CoA carboxylase (GCC) activity was first purified from a *Pseudomonas* organism grown with either the related acyclic monoterpenoid citronellol^4^ or geranic acid^5^,^6^,^7^ as the sole carbon source. GCC also possesses 3-methylcrotonyl-CoA carboxylase (MCC) activity^5^,^6^,^8^, and 3-methylcrotonyl-CoA is an intermediate in the complete catabolism of citronellol and geranic acid (Fig. 1a). In comparison, the authentic MCC enzyme cannot carboxylate the larger geranyl-CoA substrate. GCC and other enzymes involved in the first half of the geranic acid degradation pathway are encoded by the acyclic terpene utilization operon, while MCC and those involved in the second half are encoded by the leucine/isovalerate utilization operon^8^,^9^. GCC is found in *Pseudomonas* and a collection of other bacteria. MCC is conserved from bacteria to humans, and is crucial for leucine metabolism (Fig. 1a). Deficiencies in MCC activity in humans are linked to methylcrotonylglycinuria and other serious metabolic diseases^10^,^11^,^12^.

GCC and MCC are members of the biotin-dependent carboxylase superfamily^13^,^14^,^15^,^16^,^17^. Both consist of two subunits, α and β, and their holoenzymes are 750-kDa-α_6_β_6_ dodecamers. The α subunit contains the biotin carboxylase (BC), BC–CT interaction (BT)^18^,^19^, and the biotin carboxyl carrier protein (BCCP) domains (Fig. 1b, Supplementary Fig. 1). The β subunit contains the N and C domains of carboxyltransferase (CT, Supplementary Fig. 2). The α subunits of *Pseudomonas* GCC and MCC share 51% amino acid sequence identity, and their β subunits share 46% identity. These two enzymes therefore have high sequence conservation, but the molecular basis for their distinct substrate preferences is currently not known. In fact, many of the GCC/MCC enzymes in the sequence database have been misannotated (see below).

Propionyl-CoA carboxylase (PCC) shares the same domain organization as GCC and MCC (Fig. 1b). However, our recent crystal structures of MCC and PCC showed that their holoenzymes have strikingly different overall architectures^18^,^19^. Moreover, the connectivity between the N and C domains is different in the two β subunits (Supplementary Fig. 3). These results led to the hypothesis that there are two distinct lineages of biotin-dependent acyl-CoA carboxylases^19^: those that carboxylate the α carbon of the organic acid (PCC, acetyl-CoA carboxylase) and those that carboxylate the γ carbon of an α-β unsaturated acid (MCC, GCC) (Fig. 1a). A prediction of this hypothesis is that GCC should have a similar β subunit connectivity as MCC, and that the GCC holoenzyme may have a similar architecture as MCC. Here we report the crystal structure of the GCC holoenzyme, as well as mutagenesis studies to define the molecular basis for the distinct substrate preferences of GCC and MCC.

## Results

### Structure of GCC β subunit hexamer

We first determined the structure of *Pseudomonas aeruginosa* GCC (PaGCC) β subunit at 2.4 Å resolution. The current atomic model has good agreement with the crystallographic data and the expected geometric parameters (Table 1). The structure confirms our hypothesis, showing that GCCβ has the same connectivity between its N and C domains as that of MCCβ (Fig. 2a). The overall structures of the two subunits are similar, with root mean squared distance of 1.2 Å for 464 equivalent Cα atoms between them, consistent with their strong sequence conservation. The overall structures of the two β_6_ hexamers are similar to each other as well, with root mean squared distance of 1.3 Å for 2,866 equivalent Cα atoms (Fig. 2b).

However, there are regions of substantial conformational differences between the two subunits. While the connectivity in GCCβ is the same as in MCCβ, the linker between the N and C domains has a different conformation (Fig. 2a). In addition, the C-terminal segment of GCCβ (residues 526–538) contacts the N domain, while that of MCCβ contacts the C domain. The long helix at the N-terminal end of GCCβ is disordered in one of the three β subunits in the asymmetric unit (Fig. 2b), although this helix is fully ordered in the GCC holoenzyme structure. Finally, structural differences in the active site region of the two enzymes define their distinct substrate preferences (see below).

### Structure of 750-kDa GCC holoenzyme

We next determined the crystal structure at 3.1 Å resolution of the *Pseudomonas fluorescens* (also known as *P. protegens*) GCC (PfGCC) holoenzyme (Table 1, Supplementary Fig. 4). The overall architecture of the GCC holoenzyme is similar to that of MCC (Fig. 3a), consistent with our hypothesis. The GCC α subunits are arranged as trimers above and below the GCC β_6_ hexamer core (Fig. 3b), forming an elongated, cylinder-shaped holoenzyme structure (Fig. 3a). The height of the holoenzyme is ∼200 Å and the diameter of the cylinder is ∼100 Å. When the β_6_ cores of GCC free enzyme and MCC holoenzyme in complex with CoA are superposed, the BC domain in each GCC α subunit shows ∼9° difference in orientation compared with its equivalent in MCC (Fig. 3b). A similar difference is seen for the orientation and position of the BT domains in the two enzymes (Fig. 3a). In comparison, a difference of ∼6° was observed for the α subunit between the MCC free enzyme and the CoA complex^19^, and the structure of GCC free enzyme is somewhat more similar to that of the MCC free enzyme, with a difference of ∼4°. Therefore, there might be some inherent flexibility in the orientation of the α subunits relative to the β_6_ core in these holoenzymes.

The distance between the BC and CT active sites is ∼80 Å in the GCC holoenzyme (Fig. 3a). Therefore, the entire BCCP domain must translocate during GCC catalysis, as is the case with MCC (ref. 19), PCC (ref. 18), long-chain acyl-CoA carboxylase^20^, pyruvate carboxylase^21^,^22^,^23^,^24^, and urea carboxylase^25^. The BCCP-biotin is bound in the active site of the CT domain in the current structure, at roughly the same position as that in the MCC holoenzyme (Fig. 3a). In four of the six BCCP domains in the GCC holoenzyme, a portion of the structure located farthest from the holoenzyme core is disordered. In comparison, only one of the six BCCP domains is ordered in the MCC holoenzyme (Fig. 3a)^19^.

We also carried out cryo electron microscopy (cryoEM) studies on the PfGCC holoenzyme and produced a reconstruction at 5.6 Å resolution (Fig. 4a,b). The crystal structure and the cryoEM reconstruction are in excellent agreement with each other overall. The cryoEM studies therefore provide important, independent confirmation that GCC assumes the same structure in solution. The four B subdomains of BC that are disordered in the crystal are observed by cryoEM. However, the two B subdomains observed in the crystal show a different conformation in the cryoEM reconstruction. The disordered segments of the BCCP domains in the crystal are also observed by cryoEM (Fig. 4a), but the overall positions of the BCCP domains in the CT active site are essentially the same in the crystal structure and cryoEM reconstruction.

### A BT domain in the structure of GCC

The sequences of the BT domains are not well conserved among GCC, MCC and PCC, in contrast to the BC and CT domains (Supplementary Figs 1, 2). The structure of the BT domain in GCC consists of a long α-helix (αV) surrounded by an eight-stranded anti-parallel β-barrel (β22–β29, Fig. 5a). In comparison, the helix of the MCC BT domain is shorter (Fig. 5b), and its β-barrel is missing a strand (β24, Fig. 5a), although this might be unique to *P. aeruginosa* and closely related MCCs (Supplementary Fig. 1)^19^. In this regard, the structure of the GCC BT domain appears more similar to that of the PCC BT domain (Fig. 5c). On the other hand, the long loop connecting the helix to the first β strand, the ‘hook'^18^, has different conformations in the three BT domains, and mediates unique interactions that may help to determine the relative positions of the α and β subunits in these holoenzymes. These unique interactions also ensure the fidelity of each holoenzyme, such that a GCC-MCC hybrid cannot be produced.

The C-terminal segment of this hook in the GCC BT domain contains a short β-strand and establishes similar interactions with the β subunit as observed in MCC (Fig. 5a). The first residue of this β-strand is Trp483, mostly conserved among GCC and MCC enzymes (Trp543 in PaMCC, residue numbers according to human MCC, Supplementary Fig. 1). The side chain is buried at the interface between the N and C domains of the β subunit. This part of the hook is also located near the long helix (α0) at the N-terminal end of the β unit, a structural feature that is absent in PCC.

The N-terminal segment of the hook in the MCC BT domain is much longer, and establishes interactions with the linker between the N and C domains of the β subunit (Fig. 5b). In fact, the conformational differences for this linker between GCC and MCC (Fig. 2a) are likely due to this interaction with the BT domain in MCC, as the conformation of the linker in the GCC holoenzyme would clash with this part of the hook in the MCC holoenzyme (Fig. 5b). This different conformation of the linker, by up to 10 Å, is in turn coupled to the different positioning of the C-terminal segments of the β subunits. The C-terminal segments of two monomers in a β_2_ dimer are swapped with each other between GCC and MCC (Fig. 5b), again illustrating the dramatic structural plasticity among these highly conserved enzymes.

### Molecular basis for substrate preference

Based on the binding modes of CoA to MCC (ref. 19) and crotonyl-CoA to glutaconyl-CoA decarboxylase α (ref. 26), we built a model for the binding mode of geranyl-CoA in GCC (Fig. 6a). There is a large, mostly hydrophobic pocket at the bottom of the active site in GCC that can accommodate the larger geranyl group. The pocket is formed primarily by residues in helices α3, α4 and α5 in the N domain, with only minor contributions from residues in the C domain. Interestingly, the side chain of Phe191 in MCCβ is located in this pocket and clashes with the second isopentenyl group of geranyl-CoA. The equivalent residue in GCC is Gly162. There are also differences in the main-chain atoms of the residues in this region, and MCCβ contains a three-residue insertion here (Supplementary Fig. 2). Overall, these structural differences reduce the size of the active site pocket in MCCβ, thereby defining the molecular basis for why MCC is not active toward the larger geranyl-CoA substrate. Conversely, the larger pocket in GCC can accommodate both geranyl-CoA and 3-methylcrontonyl-CoA, and therefore it can be active toward both substrates.

To test these structural observations, we created the G162F mutant of GCCβ and the F191G mutant of MCCβ. The G162F mutation destabilized the GCC holoenzyme and it dissociated during gel filtration unless stabilized by higher salt concentrations. The activity of wild-type GCC toward the geranyl-CoA substrate (Fig. 6b) is about fourfold lower than that of wild-type MCC toward the 3-methylcrotonyl-CoA substrate (Fig. 6c). Wild-type GCC showed very weak activity towards 3-methylcrotonyl-CoA. The *K*_m_ of this reaction is very high, suggesting that binding of the smaller CoA compound is not optimal in the GCC active site.

As predicted by the structure, the G162F mutant of GCC is catalytically inactive toward geranyl-CoA, while the F191G mutant of MCC showed appreciable activity towards this substrate, about fivefold lower than wild-type GCC (Fig. 6b). Conversely, the G162F mutant of GCC demonstrates stronger activity towards 3-methylcrotonyl-CoA than wild-type GCC, while the F191G mutant of MCC is essentially inactive (Fig. 6c).

## Discussion

The kinetic parameters reported here for the wild-type enzymes are generally similar to those from previous studies on GCC and MCC. An apparent *K*_m_ value of 50 μM was determined from our studies on PfGCC (Fig. 6b). However, only ∼25% of the geranyl-CoA compound we used was in the *cis* configuration. Therefore, the actual *K*_m_ value is likely ∼12 μM, comparable to the 9 μM reported earlier for PaGCC (ref. 8). The *K*_m_ value for PaMCC was 69 μM based on our studies (Fig. 6c), while the earlier study reported 10 μM as well as cooperative behaviour (Hill coefficient 2.3). However, in a more recent paper^27^, the *K*_m_ was found to be 220 μM and no cooperativity was reported. These other kinetic studies are based on the CO_2_ fixation assay, while our studies are based on the coupled enzyme assay monitoring ATP hydrolysis. Some of the differences in the kinetic parameters could also be due to the differences in the assay protocols.

**Figure 1:**
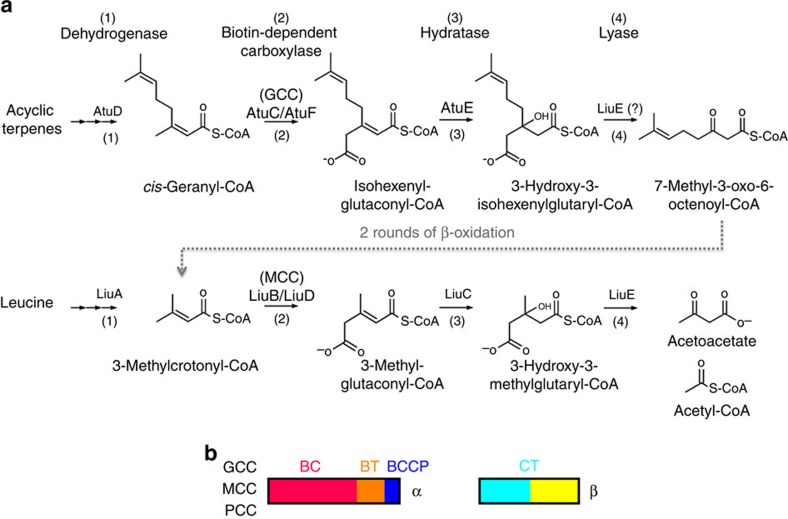
The metabolic pathways involving GCC and MCC. (**a**). The metabolism of acyclic terpenes requires the activity of both GCC and MCC, while the metabolism of the amino acid leucine requires only MCC. The Atu and Liu pathways are sequentially similar, involving dehydrogenase, carboxylase, hydratase and lyase activities. (**b**). Domain organization of GCC, MCC and PCC α and β subunits. The various domains are labeled. Atu, acyclic terpene utilization; Liu, leucine/isovalerate utilization.

**Figure 2:**
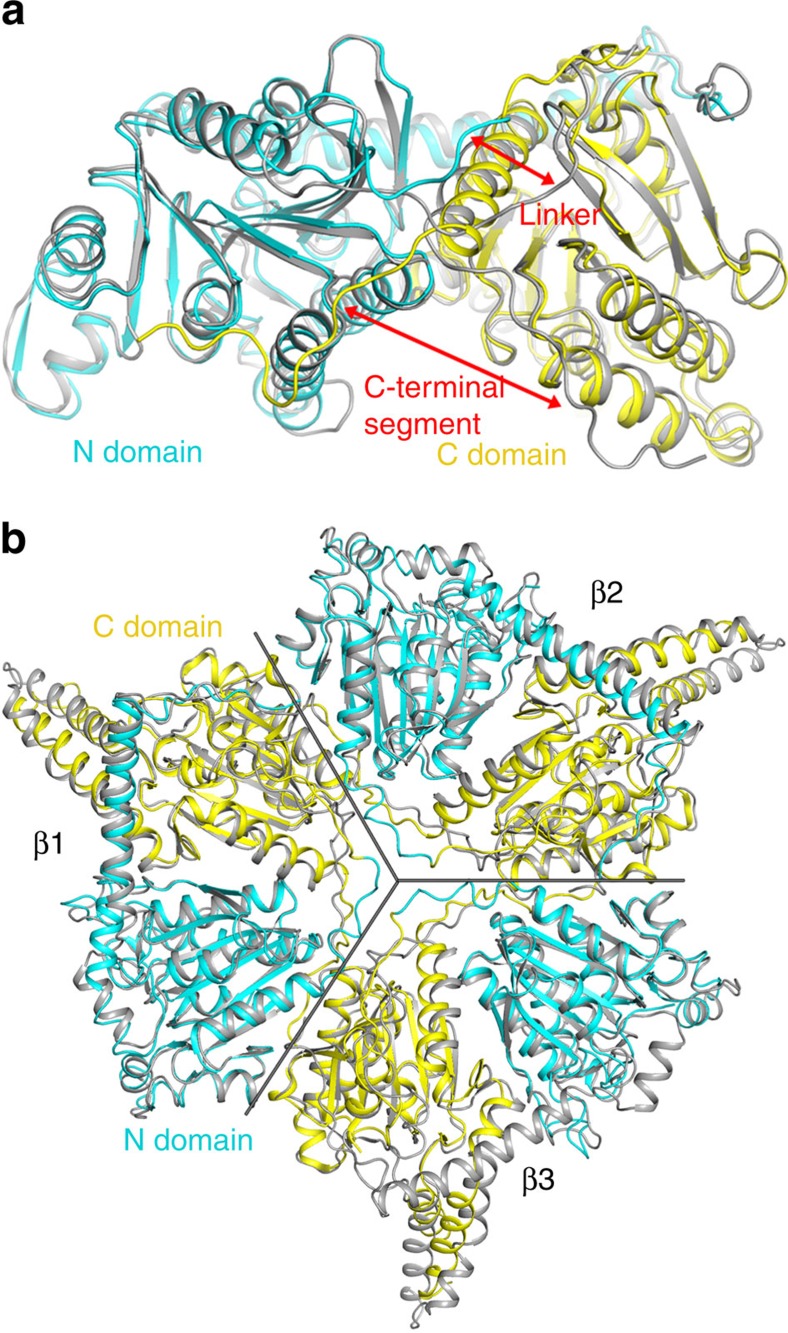
The structure of GCC β subunit. (**a**). Overlay of the structure of PaGCC β subunit (in colour) with that of PaMCC β subunit (PDB entry 3U9R, in grey)^19^. Large conformational differences for the linker between the N and C domains and the C-terminal segment are indicated with the red arrows. (**b**). Overlay of the structure of PaGCC β subunit trimer (in colour) with that of PaMCC β subunit trimer (in grey). The subunit boundaries are indicated with the grey lines. The three β subunits in the bottom layer of the β_6_ hexamer have been omitted for clarity. The long helix at the N terminus is missing in β3. All the structure figures were produced with PyMOL (www.pymol.org) unless stated otherwise.

**Figure 3:**
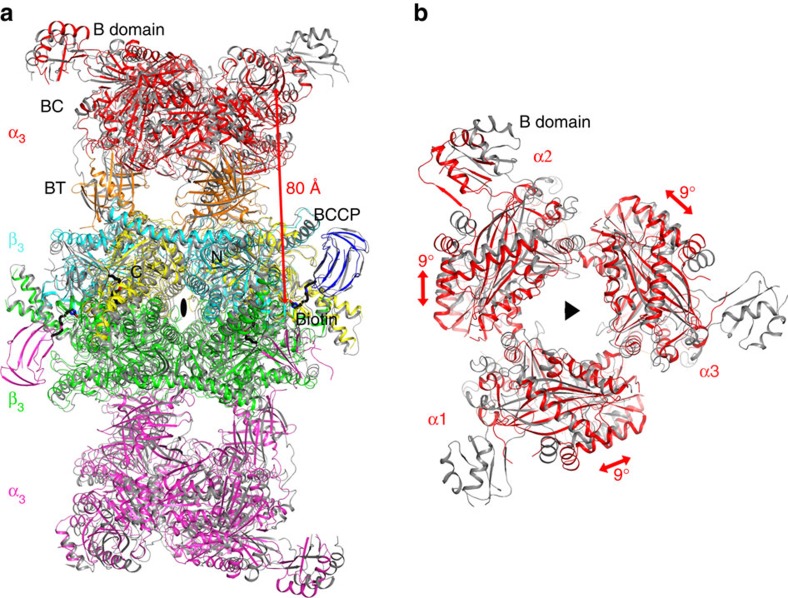
The crystal structure of the GCC holoenzyme. (**a**). Overlay of the structure of PfGCC holoenzyme (in colour) with that of PaMCC holoenzyme (PDB entry 3U9T, in grey)^19^, viewed down the two-fold symmetry axis of a β_2_ dimer (black oval). Domains in the α and β subunits in the top half of the structure are coloured as in Fig. 1b for GCC. The α and β subunits in the bottom half are coloured in magenta and green, respectively. (**b**). Overlay of the structure of three BC domains in PfGCC (in red) with that of the BC domains in PaMCC (in grey), viewed down the three-fold symmetry axis of the holoenzyme (black triangle). The differences in the conformations of the BC domains are indicated with the red arrows, corresponding to a rotation of ∼9°. Only one of the B domains of BC in the α_3_β_3_ asymmetric unit is ordered in PfGCC.

**Figure 4:**
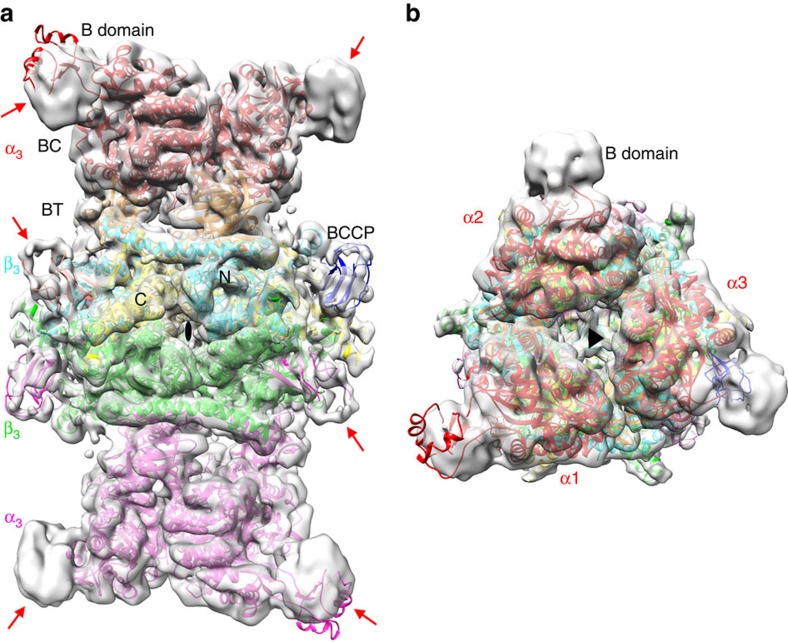
The cryo electron microscopy structure of the GCC holoenzyme. (**a**). CryoEM reconstruction of PfGCC at 5.6 Å resolution, shown as a transparent surface (in grey) and viewed in the same orientation as Fig. 3a. The crystal structure of PfGCC can be readily fit into the cryo EM density. Several of the B domains of BC and the disordered segments of BCCP in the crystal structure are observed in the cryo EM density, indicated with the red arrows. The conformation of the two ordered B domains in the crystal is somewhat different from that observed by EM. (**b**). Cryo EM reconstruction of PfGCC, viewed down the three-fold symmetry axis of the holoenzyme (black triangle). Produced with Chimera^38^.

**Figure 5:**
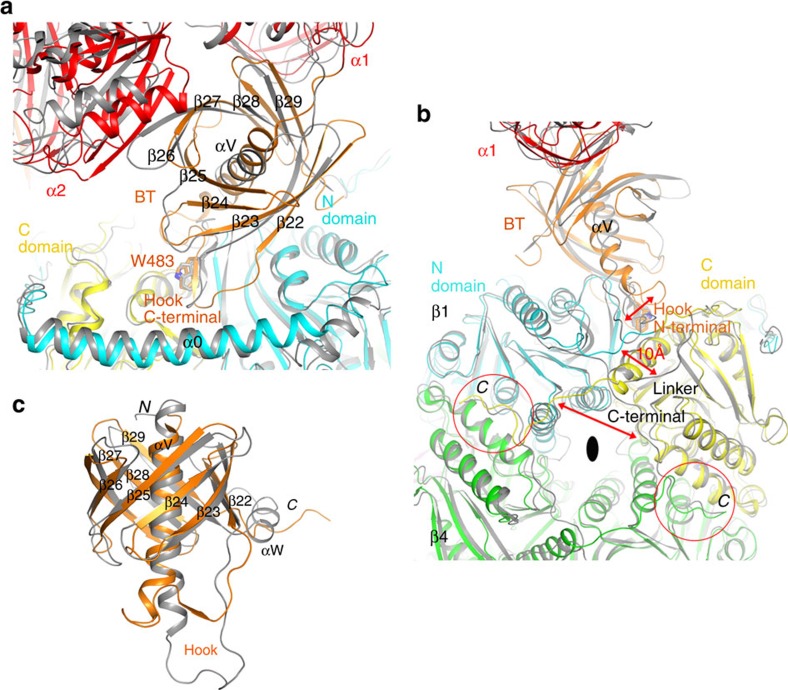
Interactions between the BT domain and the β subunit in the GCC holoenzyme. (**a**). Comparison of the interactions between the C-terminal segment of the hook in the BT domain and the β subunit in PfGCC (in colour) with those in PaMCC (in grey). Strand β24 is missing in the PaMCC BT domain, and the exact positions of many of the other β strands in the two BT domains are different as well. (**b**). Comparison of the interactions between the N-terminal segment of the hook in the BT domain and the β subunit in PfGCC (in colour) with those in PaMCC (in grey). Large conformational differences are indicated with the red arrows and labelled. The C termini of the β subunits are highlighted with the red circles. The C-terminal segments of the β_2_ dimer are swapped in GCC compared with MCC. (**c**). Overlay of the structure of GCC BT domain (in orange) with that of PCC (gray). A large conformational difference for the hook is visible.

**Figure 6:**
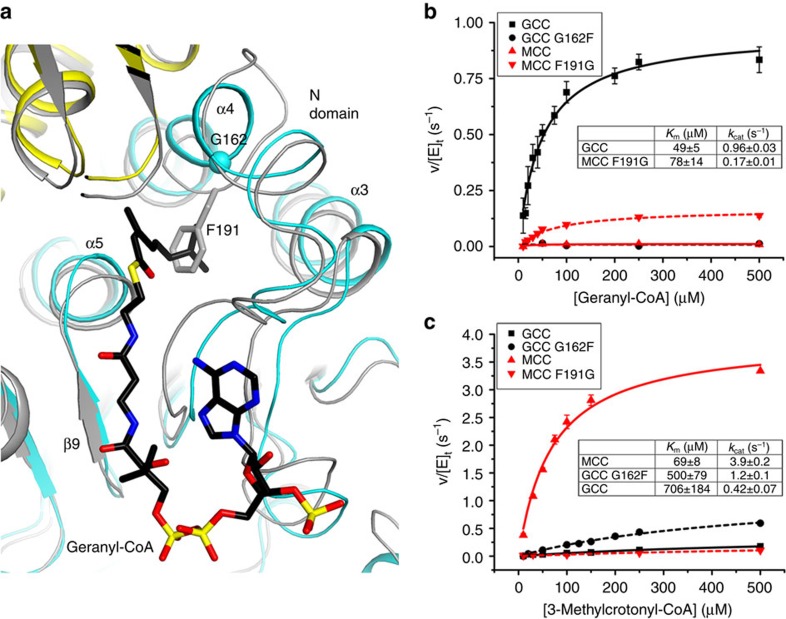
Molecular basis for the different substrate preferences of GCC and MCC. (**a**). A model for the binding mode of the geranyl-CoA substrate (in black) in the active site of GCC (cyan for the N domain and yellow for the C domain of the β subunits). The structure of MCC is superimposed and shown in grey. The side chain of Phe191 of MCCβ is shown as a stick model (grey), and the Cα atom of Gly162 of GCCβ is shown as a sphere (cyan). (**b**). Catalytic activities of wild-type GCC and MCC, the G162F mutant of GCC and the F191G mutant of MCC toward the geranyl-CoA substrate. The error bars represent the standard deviations from three separate experiments. The kinetic parameters are shown in the inset. (**c**). Catalytic activities of wild-type GCC and MCC, the G162F mutant of GCC and the F191G mutant of MCC toward the 3-methylcrotonyl-CoA substrate.

Since GCC, MCC, and PCC have similar domain organizations (Fig. 1b) and conserved sequences, we have found that they are frequently misannotated in the sequence database. The structural information on the three holoenzymes suggests guidelines for how they can be classified based on their sequences. PCCβ lacks an extended α-helix (∼30 residues) at the N terminus and can be separated from GCCβ and MCCβ based on this. The mitochondrial targeting sequence of animal PCCβ should be excluded from this consideration. MCCβ has a Phe residue in the active site region of the N domain, and this residue is replaced a Gly in GCCβ. MCCβ also has a short insertion of 3 residues just before this Phe residue compared with GCCβ. With these guidelines, it should be possible to accurately annotate the different enzymes.

Overall, our studies on GCC, MCC and PCC support the presence of two distinct lineages of biotin-dependent acyl-CoA carboxylases. The structural differences among these enzymes also have wide implications for the relationship between sequence conservation and structural similarity. The differences in substrate preferences between GCC and MCC are defined by differences in their sequences, and consequently structures, in the active site region.

## Methods

### Protein expression and purification

GCC holoenzymes were overexpressed using a bi-cistronic plasmid, with GCCα (untagged) placed downstream of GCCβ (N-terminal His-tagged) in a similar fashion to the MCC and PCC holoenzymes^18^,^19^. The hexa-histidine tag was not removed for crystallization.

PaGCC holoenzyme was overexpressed in *Escherichia* c*oli* BL21 Star (DE3) cells (Novagen) in the presence of 1 mM IPTG (Gold Biotechnology, Inc) to induce expression and incubated at 20 °C overnight. The soluble protein was eluted from Ni-NTA beads (Qiagen) and further purified by gel filtration in a column buffer containing 25 mM Tris (pH 7.4), 250 mM NaCl and 2 mM DTT. The purified protein was concentrated to 12 mg ml^−1^, supplemented with 5% (v/v) glycerol, flash-frozen with liquid nitrogen and stored at −80 °C. An SDS gel of this sample showed that the α subunit was present in significantly substoichiometric amounts.

*P. fluorescens* GCC (PfGCC) holoenzyme was expressed in the same condition as the PaGCC holoenzyme and purified by gel filtration in a column buffer containing 20 mM Tris (pH 8.5), 200 mM NaCl and 10 mM DTT. The purified protein was concentrated to 20 mg ml^−1^, supplemented with 5% (v/v) glycerol, flash-frozen with liquid nitrogen and stored at −80 °C. An SDS gel of this sample showed stoichiometric amounts of both subunits.

### Protein crystallization

Crystals of PaGCC β_6_ hexamer were obtained by using the PaGCC holoenzyme protein sample (but with significantly substoichiometric amounts of the α subunit) with the hanging-drop vapour diffusion method. Crystals of PaGCC β subunit appeared after 2 weeks at 20 °C from a precipitant solution containing 100 mM Tris (pH 8.0) and 60% (v/v) MPD. Crystals were flash-frozen in liquid nitrogen for data collection at 100 K. Interestingly, crystals obtained by using a pure PaGCC β_6_ sample diffracted very poorly.

Crystals of the PfGCC holoenzyme were obtained using the microbatch-under-oil method at 20 °C. The protein (20 mg ml^−1^) was mixed with the precipitant solution at a ratio of 2:1, which contained 100 mM Tris (pH 7.0), 2 M (NH_4_)_2_SO_4_ and 250 mM Li_2_SO_4_. Crystals appeared after 1 month, and were cryoprotected by supplementing the precipitant solution with 30% (v/v) ethylene glycol.

### Data collection and structure determination

X-ray diffraction data sets were collected on an ADSC Q315 CCD at the X29A beamline and on a Pilatus 6 M detector at the X25 beamline of the National Synchrotron Light Source at the Brookhaven National Laboratory. The diffraction images were processed using the HKL package^28^. The data processing and refinement statistics are summarized in Table 1. The resolution limit for the PfGCC holoenzyme data was determined using the CC_1/2_ criterion^29^.

Crystals of PaGCCβ contained one β_3_ trimer in the asymmetric unit. The structure was determined by the molecular replacement method with the program Phaser^30^, using the structure of MCCβ as the model^19^. Crystals of the PfGCC holoenzyme contained half of the dodecamer (α_3_β_3_) in the asymmetric unit. The structure was determined with the molecular replacement method using GCCβ_3_ and MCCα_3_ as the search models. Structure refinement was carried out with the programs CNS^31^ and Refmac^32^, and the program Coot^33^ was used for manual model rebuilding.

### CryoEM imaging

To prepare cryoEM grids, we applied 2 μl of purified PfGCC sample to thin continuous carbon films on lacey grids (Ted Pella Inc.) for 1 min, blotted for 12 s under 100% humidity and plunged the grid into liquid ethane with an FEI Vitrobot (ambient temperature in the Vitrobot chamber was 20 °C). CryoEM images were collected at liquid nitrogen temperature in an FEI Titan Krios cryo electron microscope operated at 300 kV using parallel illumination. Before data collection, the microscope was carefully aligned, and beam tilt was minimized by coma-free alignment. Images were recorded on a Gatan K2 camera with the counting mode at a nominal magnification of × 29,000 on microscope. The magnification on the camera was calibrated as × 49,500 using a catalase crystal sample, giving a pixel size of 1.01 Å per pixel on specimen. The dose rate of the electron beam was set to ∼8 counts per pixel per s on the camera which corresponds to ∼10 e^−^ Å^−2^ s^−1^ on specimen when including the electrons uncounted by the K2 camera. Image stacks were recorded at 4 frames per sec for 10 s. After drift correction with the UCSF software^34^, the first 12 frames of each image stack (movie) were merged to generate a final image with a total dose of ∼30 e^−^ Å^−2^ of the sample.

### CryoEM image processing

A total of 41,271 particle images were picked from 1,149 images automatically with the *Dog Picker* program^35^. The under defocus values of these images were determined to be between 1.06 to 3.4 μm using CTFFIND^36^. Image processing and reconstruction were carried out using RELION^37^. Two-dimensional classification was first used to screen particles. After 25 iterations, 16 two-dimensional classes with 31,206 particles were picked from the total 30 classes. Three-dimensional (3D) classification was then performed with RELION to classify all particles into three groups. However, the reconstructions from these three groups are nearly identical. We therefore used all 31,206 particles for 3D refinement, which started from a featureless starting model generated by low-pass (60 Å resolution) filtering the structure of MCC (PDB code: 3U9T; ref. 19). The 32 symmetry of the particles was also applied in the calculation. After 16 cycles of 3D refinement, the resolution of GCC structure was estimated to be ∼5.6 Å using the ‘gold-standard FSC' criterion with RELION. The density map was sharpened with a reverse B-factor of −250 Å^2^. Visualization and segmentation of density maps were carried out with UCSF Chimera^38^.

### Mutagenesis and kinetic studies

Site-specific mutations in PfGCC and PaMCC were introduced with the QuikChange kit (Agilent Technologies) and expressed and purified under the same conditions as PfGCC (gel filtration buffer 20 mM Tris (pH 8.5), 200 mM NaCl, 5 mM DTT).

The catalytic activity of PaMCC, PfGCC and their mutants was determined using a coupled enzyme assay, converting the hydrolysis of ATP to the disappearance of NADH (refs 39, 40). The reaction mixture contained 100 mM HEPES (pH 8.0), 0.5 mM ATP, 8 mM MgCl_2_, 40 mM KHCO_3_, 2–500 μM 3-methylcrotonyl-CoA or crotonyl-CoA, 0.2 mM NADH, 0.5 mM phosphoenolpyruvate, 7 units of lactate dehydrogenase, 4.2 units of pyruvate kinase and 250 mM KCl. The absorbance at 340 nm was monitored for 5 min. Geranyl-CoA was synthesized chemically from geranic acid and CoA (Changchun Discovery Sciences, Ltd), and contained ∼25% *cis* isomer based on HPLC and proton NMR measurements. Based on the structure, the *trans* isomer is unlikely to be accommodated in the active site, and therefore could not serve as a substrate or an inhibitor of the enzyme.

## Additional information

**How to cite this article**: Jurado, A. R. *et al*. Structure and substrate selectivityof the 750-kDa α_6_β_6_ holoenzyme of geranyl-CoA carboxylase. *Nat. Commun.* 6:8986 doi: 10.1038/ncomms9986 (2015).

## Supplementary Material

Supplementary Information Supplementary Figures 1-4 and Supplementary Reference

## Figures and Tables

**Table 1:** Summary of crystallographic information.

	PaGCCβ hexamer	PfGCC holoenzyme
*Data collection*		
Space group	*P*6_5_22	*P*4_1_2_1_2
Cell dimensions		
*a*, *b*, *c* (Å)	105.7, 105.7, 550.8	234.6, 234.6, 274.4
α, β, γ (°)	90, 90, 120	90, 90, 90
Resolution (Å)*	30–2.4 (2.53–2.4)	50–3.1 (3.26–3.1)
*R*_merge_	8.4 (43.2)	12.2 (>100)
CC_1/2_	–	0.48
*I*/σ*I*	15.4 (3.1)	9.7 (1.0)
Completeness (%)	100 (99)	98 (95)
Redundancy	4.5 (4.5)	3.7 (3.6)
		
*Refinement*		
Resolution (Å)	47–2.4	50–3.1
No. of reflections	68,848	128,390
*R*_work_/*R*_free_	21.6/26.2	19.6/22.8
No. of atoms		
Protein	11,411	25,281
Ligand/ion	0	0
Water	172	0
*B*-factors		
Protein	66	99
Ligand/ion	–	–
Water	52	–
R.m.s. deviations		
Bond lengths (Å)	0.013	0.011
Bond angles (°)	1.6	1.5

PaGCC, *Pseudomonas aeruginosa* geranyl-CoA carboxylase; PfGCC, *Pseudomonas fluorescens* geranyl-CoA carboxylase; r.m.s., root mean squared.

^*^The numbers in parentheses are for the highest resolution shell.

## References

[b1] Thulasiram H. V., Erickson H. K. & Poulter C. D. Chimeras of two isoprenoid synthases catalyze all four coupling reactions in isoprenoid biosynthesis. Science 316, 73–76 (2007). 17412950 10.1126/science.1137786

[b2] Vranova E., Coman D. & Gruissern W. Network analysis of the MVA and MEP pathways for isoprenoid synthesis. Annu. Rev. Plant Biol. 64, 665–700 (2013). 23451776 10.1146/annurev-arplant-050312-120116

[b3] Rodriguez-Concepcion M. Plant isoprenoids: a general overview. Methods Mol. Biol. 1153, 1–5 (2014). 24777786 10.1007/978-1-4939-0606-2_1

[b4] Seubert W., Fass E. & Remberger U. Studies on the bacterial degradation of isoprenoid compounds. III. Purification and properties of geranyl carboxylase. Biochem. Z. 338, 265–275 (1963). 14087299

[b5] Hector M. L. & Fall R. R. Multiple acyl-coenzyme A carboxylases in *Pseudomonas citronellolis*. Biochemistry 15, 3465–3472 (1976). 8091 10.1021/bi00661a011

[b6] Hector M. L. & Fall R. R. Evidence for distinct 3-methylcrotonyl-CoA and geranyl-CoA carboxylases in *Pseudomonas citronellolis*. Biochem. Biophys. Res. Commun. 71, 746–753 (1976). 962953 10.1016/0006-291x(76)90894-9

[b7] Fall R. R. & Hector M. L. Acyl-coenzyme A carboxylases. Homologous 3-methylcrotonyl-CoA and geranyl-CoA carboxylase from *Pseudomonas citronellolis*. Biochemistry 16, 4000–4005 (1977). 911753 10.1021/bi00637a010

[b8] Aguilar J. A. . Substrate specificity of the 3-methylcrotonyl coenzyme A (CoA) and geranyl-CoA carboxylases from *Pseudomonas aeruginosa*. J. Bacteriol. 190, 4888–4893 (2008). 18469096 10.1128/JB.00454-08 PMC2447018

[b9] Forster-Fromme K. & Jendrossek D. Catabolism of citronellol and related acyclic terpenoids in pseudomonads. Appl. Microbiol. Biotechnol. 87, 859–869 (2010). 20490788 10.1007/s00253-010-2644-x

[b10] Baumgartner M. R. . The molecular basis of human 3-methylcrotonyl-CoA carboxylase deficiency. J. Clin. Invest. 107, 495–504 (2001). 11181649 10.1172/JCI11948 PMC199271

[b11] Gallardo M. E. . The molecular basis of 3-methylcrotonylglycinuria, a disorder of leucine metabolism. Am. J. Hum. Genet. 68, 334–346 (2001). 11170888 10.1086/318202 PMC1235267

[b12] Holzinger A. . Cloning of the human MCCA and MCCB genes and mutations therein reveal the molecular cause of 3-methylcrotonyl-CoA carboxylase deficiency. Hum. Mol. Genet. 10, 1299–1306 (2001). 11406611 10.1093/hmg/10.12.1299

[b13] Wakil S. J., Stoops J. K. & Joshi V. C. Fatty acid synthesis and its regulation. Annu. Rev. Biochem. 52, 537–579 (1983). 6137188 10.1146/annurev.bi.52.070183.002541

[b14] Tong L. Structure and function of biotin-dependent carboxylases. Cell. Mol. Life Sci. 70, 863–891 (2013). 22869039 10.1007/s00018-012-1096-0 PMC3508090

[b15] Cronan J. E. Jr. & Waldrop G. L. Multi-subunit acetyl-CoA carboxylases. Prog. Lipid Res. 41, 407–435 (2002). 12121720 10.1016/s0163-7827(02)00007-3

[b16] Jitrapakdee S. . Structure, mechanism and regulation of pyruvate carboxylase. Biochem. J. 413, 369–387 (2008). 18613815 10.1042/BJ20080709 PMC2859305

[b17] Nikolau B. J., Ohlrogge J. B. & Wurtele E. S. Plant biotin-containing carboxylases. Arch. Biochem. Biophys. 414, 211–222 (2003). 12781773 10.1016/s0003-9861(03)00156-5

[b18] Huang C. S. . Crystal structure of the a6b6 holoenzyme of propionyl-coenzyme A carboxylase. Nature 466, 1001–1005 (2010). 20725044 10.1038/nature09302 PMC2925307

[b19] Huang C. S., Ge P., Zhou Z. H. & Tong L. An unanticipated architecture of the 750-kDa a6b6 holoezyme of 3-methylcrotonyl-CoA carboxylase. Nature 481, 219–223 (2012). 22158123 10.1038/nature10691 PMC3271731

[b20] Tran T. H. . Structure and function of a single-chain, multi-domain long-chain acyl-CoA carboxylase. Nature 518, 120–124 (2015). 25383525 10.1038/nature13912 PMC4319993

[b21] St. Maurice M. . Domain architecture of pyruvate carboxylase, a biotin-dependent multifunctional enzyme. Science 317, 1076–1079 (2007). 17717183 10.1126/science.1144504

[b22] Xiang S. & Tong L. Crystal structures of human and Staphylococcus aureus pyruvate carboxylase and molecular insights into the carboxyltransfer reaction. Nat. Struct. Mol. Biol. 15, 295–302 (2008). 18297087 10.1038/nsmb.1393

[b23] Yu L. P. C. . A symmetrical tetramer for S. aureus pyruvate carboxylase in complex with coenzyme A. Structure. 17, 823–832 (2009). 19523900 10.1016/j.str.2009.04.008 PMC2731552

[b24] Lasso G. . Functional conformations for pyruvate carboxylase during catalysis explored by cryoEM. Structure 22, 911–922 (2014). 24882745 10.1016/j.str.2014.04.011 PMC4090597

[b25] Fan C., Chou C.-Y., Tong L. & Xiang S. Crystal structure of urea carboxylase provides insights into the carboxyltransfer reaction. J. Biol. Chem. 287, 9389–9398 (2012). 22277658 10.1074/jbc.M111.319475 PMC3308809

[b26] Kress D. . An asymmetric model for Na+-translocating glutaconyl-CoA decarboxylase. J. Biol. Chem. 284, 28401–28409 (2009). 19654317 10.1074/jbc.M109.037762 PMC2788889

[b27] Diaz-Perez C., Diaz-Perez A. L., Rodriguez-Zavala J. S. & Campos-Garcia J. Structural evidence for the involvement of the residues Ser187 and Tyr422 in substrate recognition in the 3-methylcrotonyl-coenzyme A carboxylase from *Pseudomonas aeruginosa*. J. Biochem. 154, 291–297 (2013). 23760555 10.1093/jb/mvt055

[b28] Otwinowski Z. & Minor W. Processing of X-ray diffraction data collected in oscillation mode. Method Enzymol. 276, 307–326 (1997). 10.1016/S0076-6879(97)76066-X 27754618

[b29] Karplus P. A. & Diederichs K. Linking crystallographic model and data quality. Science 336, 1030–1033 (2012). 22628654 10.1126/science.1218231 PMC3457925

[b30] McCoy A. J. . Phaser crystallographic software. J. Appl. Cryst. 40, 658–674 (2007). 19461840 10.1107/S0021889807021206 PMC2483472

[b31] Brunger A. T. . Crystallography & NMR System: a new software suite for macromolecular structure determination. Acta Crystallogr. D Biol. Crystallogr. 54, 905–921 (1998). 9757107 10.1107/s0907444998003254

[b32] Murshudov G. N., Vagin A. A. & Dodson E. J. Refinement of macromolecular structures by the maximum-likelihood method. Acta Crystallogr. D Biol. Crystallogr. 53, 240–255 (1997). 15299926 10.1107/S0907444996012255

[b33] Emsley P. & Cowtan K. D. Coot: model-building tools for molecular graphics. Acta Crystallogr. D Biol. Crystallogr. 60, 2126–2132 (2004). 15572765 10.1107/S0907444904019158

[b34] Li X. . Electron counting and beam-induced motion correction enable near-atomic-resolution single-particle cryo-EM. Nat. Methods 10, 584–590 (2013). 23644547 10.1038/nmeth.2472 PMC3684049

[b35] Voss N., Yoshioka C., Radermacher M., Potter C. & Carragher B. DoG Picker and TiltPicker: software tools to facilitate particle selection in single particle electron microscopy. J. Struct. Biol. 166, 205–213 (2009). 19374019 10.1016/j.jsb.2009.01.004 PMC2768396

[b36] Mindell J. A. & Grigorieff N. Accurate determination of local defocus and specimen tilt in electron microscopy. J. Struct. Biol. 142, 334–347 (2003). 12781660 10.1016/s1047-8477(03)00069-8

[b37] Scheres S. H. RELION: implementation of a Bayesian approach to cryo-EM structure determination. J. Struct. Biol. 180, 519–530 (2012). 23000701 10.1016/j.jsb.2012.09.006 PMC3690530

[b38] Pettersen E. F. . UCSF Chimera-a visualization system for exploratory research and analysis. J. Comput. Chem. 25, 1605–1612 (2004). 15264254 10.1002/jcc.20084

[b39] Diacovich L. . Crystal structure of the b-subunit of acyl-CoA carboxylase: structure-based engineering of substrate specificity. Biochemistry 43, 14027–14036 (2004). 15518551 10.1021/bi049065v

[b40] Blanchard C. Z., Lee Y. M., Frantom P. A. & Waldrop G. L. Mutations at four active site residues of biotin carboxylase abolish substrate-induced synergism by biotin. Biochemistry 38, 3393–3400 (1999). 10079084 10.1021/bi982660a

