# Supplementary material for: Structure and substrate selectivity of the 750-kDa α6β6 holoenzyme of geranyl-CoA carboxylase
Source: Nat Commun. 2015 Nov 23;6:8986. doi: 10.1038/ncomms9986 (PMC4673880; doi:10.1038/ncomms9986)
Supplement: Supplementary Information — Supplementary Figures 1-4 and Supplementary Reference [file ncomms9986-s1.pdf]

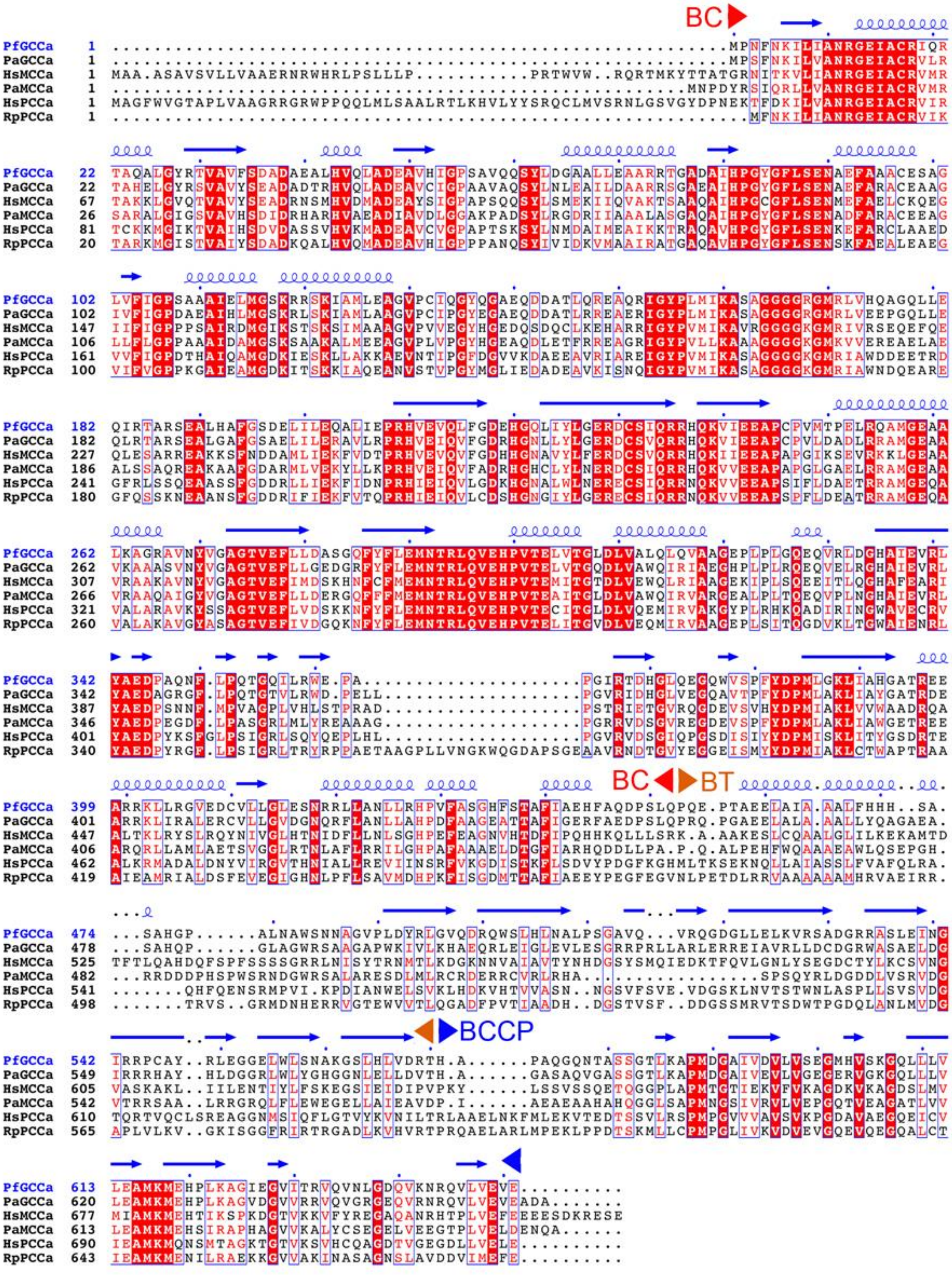

Supplementary Figure 1. Sequence alignment of the α subunits of *P. fluorescens* GCC (PfGCC), *P. aeruginosa* GCC (PaGCC), human MCC (HsMCC), PaMCC, HsPCC, and *R. pomeroyi* PCC (RpPCC). The BC, BT, and BCCP domains are indicated. The dots at the top of the alignment mark every 10th residue in PfGCC. Modified from an output from ESPript

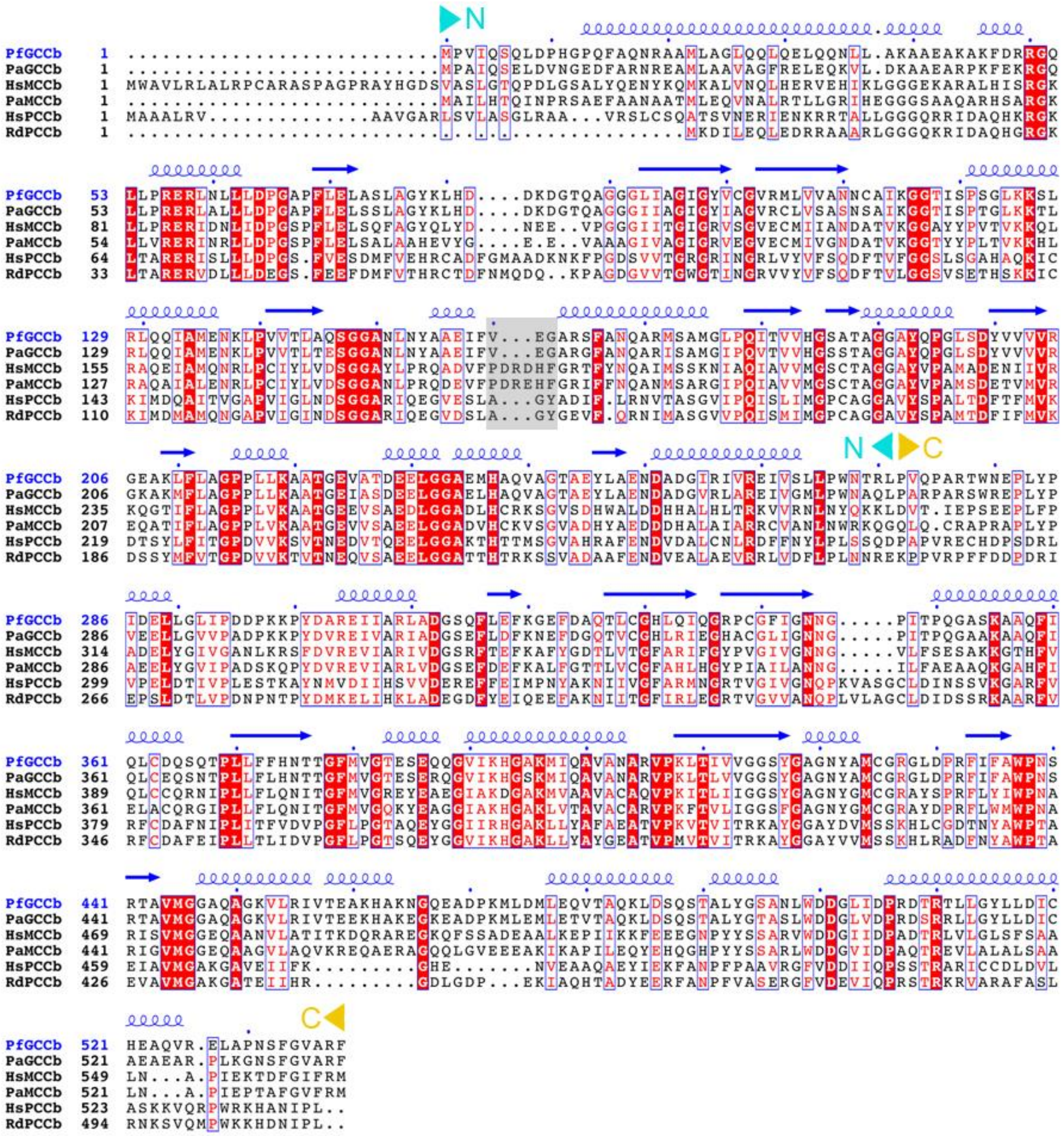

Supplementary Figure 2. Sequence alignment of the β subunits of *P. fluorescens* GCC (PfGCC), *P. aeruginosa* GCC (PaGCC), human MCC (HsMCC), PaMCC, HsPCC, and *R. denitrificans* PCC (RdPCC). The N and C domains are indicated. Residues near the binding site for the geranyl group are highlighted in gray. The dots at the top of the alignment mark every 10th residue in PfGCC.

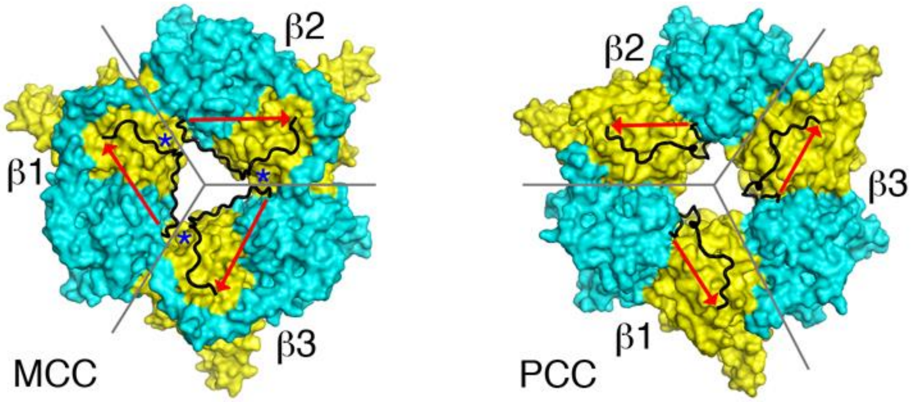

Supplementary Figure 3. Different connectivity of the N and C domains in MCC *versus* PCC β subunit. The linker from the N (cyan) to the C (yellow) domains (in black, with the red arrow indicating the direction of linker) runs in opposite directions in MCC (*Left*) and PCC (*Right*) and connects to different C domains, leading to the swapping of the positions of the N and C domains in each subunit between the two enzymes. The two neighboring linkers approach each other closely at one point (blue star) in PaMCC, and a change in connectivity at that position will lead to the PCC organization. The boundaries of each subunit are indicated by the gray lines.

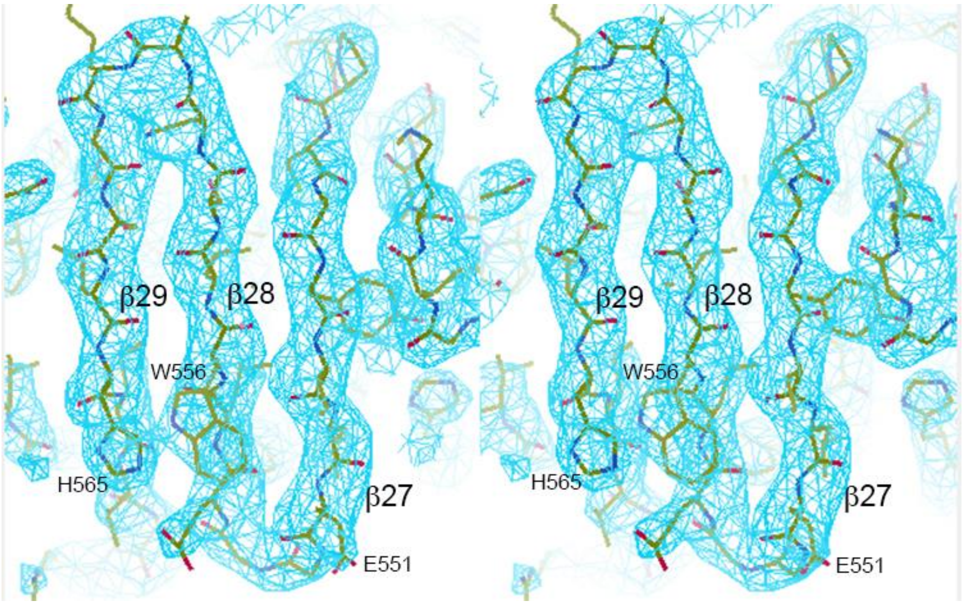

Supplementary Figure 4. Electron density for GCC holoenzyme. Stereo drawing showing the final $2F_o–F_c$ electron density at 3.1 Å resolution for the last three strands (β27-β29) of the BT domain in the structure of the GCC holoenzyme, contoured at 1.7σ.

Supplementary Reference

1. Gouet, P., Courcelle, E., Stuart, D. I. & Metoz, F. ESPript: analysis of multiple sequence alignments in PostScript. *Bioinformatics* **15**, 305-308 (1999).
